# What factors contribute to maintaining mental well-being during the COVID-19 pandemic? Corinna Kausmann

**DOI:** 10.1093/eurpub/ckac131.067

**Published:** 2022-10-25

**Authors:** C Kausmann, C Cohrdes, RKI-SOEP-2-study group

**Affiliations:** 1 Epidemiology and Health Monitoring, Robert Koch Institute, Berlin, Germany; 2 Socio-Economic Panel, German Institute for Economic Research, Berlin, Germany; 3 Federal Office for Migration and Refugees, Nuremberg, Germany; 4 Institute for Employment Research, Nuremberg, Germany

## Abstract

**Background:**

The COVID-19 pandemic posed numerous challenges for many people and first results indicate high variability of responses in mental well-being. Thus, this research aimed to identify longitudinal factors contributing to the maintenance of mental well-being despite the adverse life circumstances and derive recommendations suitable for the promotion of mental well-being in the context of future pandemic or similar stressful situations.

**Methods:**

We analysed representative longitudinal panel data of the Socio-Economic Panel (SOEP) from 15,122 adults (age range 18-99 years) who participated in the collaborative SOEP-CoV and RKI-SOEP surveys comprising self-reports of mental well-being (e.g., life satisfaction) and potentially relevant factors (e.g., control beliefs). By taking data from before (2015-2019) and during the COVID-19 pandemic (2020-2021) into account, we investigated different patterns of mental well-being trajectories and factors associated with the maintenance of mental well-being over time.

**Results:**

Preliminary results suggest that the majority of adults in Germany managed to maintain or even enhance their mental well-being in the considered time frame. Moreover, results suggest that certain factors seem to be of universal importance (e.g., altruism, locus of control) while others are particularly relevant for distinct mental well-being dimensions (e.g., life goals). Most decisively, the probability of experiencing mental well-being deterioration during the COVID-19 pandemic was enhanced in individuals with an internal locus of control.

**Conclusions:**

The findings revealed longitudinal factors that contribute to maintaining mental well-being during the COVID-19 pandemic. Promoting altruism, family-related life goals and external locus of control beliefs can help fostering resilient responses in the face of challenging life events such as the COVID-19 pandemic.

**Key messages:**

• Several factors (e.g., locus of control) offer the potential to maintain or even improve mental well-being in times characterized by the COVID-19 pandemic.

• To enhance preparedness for stressful life events such as the COVID-19 pandemic, the identified key factors should be included in basic universal public health promotion.

